# Effects of tocotrienol on aging skin: A systematic review

**DOI:** 10.3389/fphar.2022.1006198

**Published:** 2022-10-10

**Authors:** Nur Izyani Ghazali, Rahimah Zahidah Mohd Rais, Suzana Makpol, Kok Yong Chin, Wei Ney Yap, Jo Aan Goon

**Affiliations:** ^1^ Department of Biochemistry, Faculty of Medicine, Universiti Kebangsaan, Kuala Lumpur, Malaysia; ^2^ Department of Pharmacology, Faculty of Medicine, Universiti Kebangsaan, Kuala Lumpur, Malaysia; ^3^ Research and Development Department, Davos Life Science, Singapore, Singapore

**Keywords:** tocotrienol, vitamin E, skin, ultraviolet, pigmentation, moisturisation, wrinkle, aging

## Abstract

The skin is the largest organ of the body that protects from mechanical, thermal, and physical injury. However, the function and appearance of skin visibly degenerates with age due to its frequent exposure to harmful effects of the environment, including ultraviolet irradiation and hazardous substances, in addition to the progression of oxidative stress in aging. These factors result in phenotypic changes in the skin, including wrinkling, pigmentation, reduced elasticity, and hydration during aging. Many natural antioxidant compounds have been studied extensively to reverse the signs of aging skin. Tocotrienols are a subfamily of vitamin E with potent antioxidant activity. Therefore, supplementation with vitamin E in the form of tocotrienol may efficiently protect skin from aging. In this review, the effects of tocotrienol on skin health, including pigmentation, moisture, and wrinkles during aging and UV exposure, were systematically evaluated based on a literature search of the PubMed and Scopus databases. The present data showed that tocotrienols protect the skin from inflammation, UV radiation and melanin accumulation. As the therapeutic value of tocotrienols grows, the potential of these vitamin E analogs to the skin requires further investigation.

## Introduction

The skin is the primary protection layer that shields the body from environmental effects (Cravello and Ferri, 2008). However, the function of the skin deteriorates with age at a rate that is influenced by lifestyle (Levakov et al., 2012). The characteristics of degenerative skin mechanical properties include declining skin elasticity, depletion of collagen content, higher wrinkle visibility and increased pigmentation (Ryu et al., 2008). In addition, aging also alters physiological aspects of the skin, such as transepidermal water loss (TEWL), diminished skin hydration and sebum secretion (Luebberding et al., 2013).

Skin aging involves changes that occur in the lower dermis of the skin as well as depletion of subcutaneous fat over time. Ultraviolet (UV) radiation is one of the main factors that induces skin aging by breaking down connective tissues of the skin, such as collagen and elastin. UV also causes skin damage through DNA photodamage and reactive oxygen species (ROS) generation. Ultimately, skin damage manifests through two mechanisms, namely, photoaging and photocarcinogenesis. Photoaging is the damage to skin caused by UV radiation (Gu et al., 2020; Chaiprasongsuk and Panich, 2022), whereas photocarcinogenesis refers to the development of skin cancer as a result of UV radiation (Martens et al., 2018).

When DNA damage occurs as a result of UV radiation, repair mechanisms will switch on to oppose the development of mutations and skin carcinogenesis. If the repair mechanism fails to conduct its role, it will cause the inactivation of tumor-suppressor genes and elimination of negative regulatory proteins that subsequently lead to tumor development. Oxidative stress induced by UV can overwhelm the cellular antioxidant defense, damage cellular structural macromolecules and ultimately damage cells responsible for skin strength, resilience and stability (Rinnerthaler et al., 2015; Gu et al., 2020). The skin damage induced by UV radiation can manifest in several forms, such as wrinkles, skin laxity, a leathery appearance, sensitivity, impaired wound healing, and increased skin vulnerability.

**FIGURE 1:**
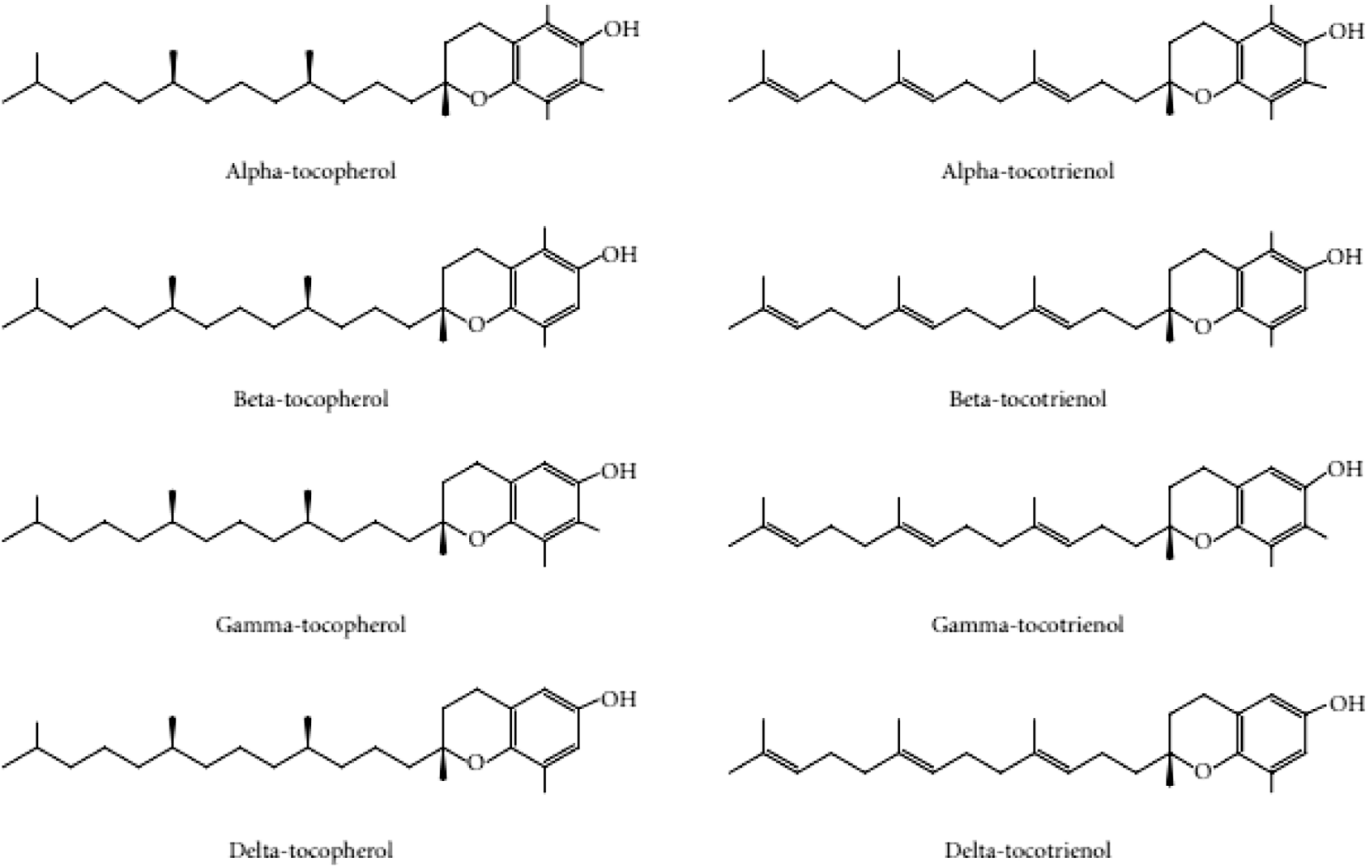
Tocopherol and tocotrienol structure adapted with permission from Chin and Ima-Nirwana (2012).

Given the damaging effects of oxidative stress on skin aging, the use of antioxidants to reverse the degenerative effects of the skin is a promising approach to tackle this condition. Vitamin E is a compound of interest and active ingredient in anti-aging cosmetic products (Brownlow et al., 2015). The Vitamin E family consists of tocotrienol and tocopherol isomers. Tocopherols are saturated forms of vitamin E, whereas tocotrienols are unsaturated with an isoprenoid side chain. Both tocopherols and tocotrienols exist in four different isoforms (-α, -β, -ɣ, and -δ), which differ in the position of the methyl groups on the chromanol ring (Packer et al., 2001) (Figure 1). Both families of vitamin E have similar functions in scavenging free radicals in cells due to their chromanol ring structure. This structure allows them to regulate signaling pathways, such as inflammation, cell apoptosis and proliferation (Wallert et al., 2020). The presence of the prenyl side chain in tocotrienol increases its permeability through the cell membranes and uptake of unstable electrons from free radicals (Bardhan et al., 2011).

Botanical oils derived from oil palm, rice bran, and annatto bean are rich in tocotrienols (Lester et al., 2001). Compared to tocopherol isoforms, tocotrienols are assimilated into cellular membranes more easily and absorbed by skin more effectively (Makpol et al., 2010). Previous studies found that the dietary supplementation (Yamada et al., 2008) and topical administration (Traber et al., 1997) of tocotrienols successfully increased the concentration of its isomers in the skin, which facilitates its function as a skin protective agent. In contrast, vitamin C, which is widely used in skincare products, is repelled by terminally differentiated epidermal cells due to its water solubility (Pullar et al., 2017). Vitamin C in the form of ascorbic acid can only penetrate the skin when the pH level of the skin is below 4 (Pinnell, 2003). These findings suggest that tocotrienols may provide more protection to aging skin than tocopherols and vitamin C. Thus, this review aims to summarize the effects of tocotrienols on age-related skin conditions based on evidence from *in vitro* and *in vivo* studies.

## Methods

### Literature search

The literature search, data extraction and systematic review were undertaken following the principles of the PRISMA guidelines. The literature search was performed in July 2022. Original research studies published in English were selected following a computer-assisted literature search of two online databases, i.e., PubMed and Scopus. The search used a combination of keywords related to the aim of this review: tocotrienol and skin.

### Article selection

The relevance of studies was assessed using a hierarchical approach based on the title, abstract, and the full manuscript. The inclusion criteria for selection were 1) original research articles and 2) studies using cell culture, animals or human subjects. The exclusion criteria were 1) review articles, books and conference abstracts and 2) articles written in languages other than English. To assist in organizing the search results, the Endnote software (version 20, Clarivate, Philadelphia, United States) was used. Duplicates were also removed using Endnote, and the results were cross-checked manually.

### Article screening

Three authors performed the search individually and screened items based on titles and abstracts. The initial screening results were discussed, and any discrepancy was resolved with discussion. The full texts of the agreed items were retrieved and screened. Data extracted from the full-text articles included names of authors, year of publication, study design and major findings of the study.

## Results

A total of 221 articles were obtained from both databases, with 70 articles from PubMed and 151 papers from Scopus. A total of 103 duplicates were removed, which resulted in 118 unique articles. Of these papers, 100 were excluded based on the selection criteria (48 were reviews, 1 was a book chapter, 2 were conference abstracts, and 49 were on irrelevant topics). A total of 18 articles were reviewed.

### Tocotrienol and skin aging

Five studies investigated the effects of tocotrienol on skin aging (Table 1). In a study by Makpol et al. (2010), normal skin fibroblast cell lines derived from adults of different ages (21, 41, and 68 years old) were used. In 3 other studies by the group, circumcised foreskin samples from male children between the ages of 9 and 12 years old were used. The circumcised foreskins were induced with oxidative stress to achieve premature senescence. In another study by Nagakawa et al. (2010), immortalized keratinocytes stimulated by squalene hyperoxide were used. All studies used γ-tocotrienol in the range of 0.1–80 µM. The outcomes of aging measured included collagen production/degradation, apoptosis and inflammation.

**TABLE 1:** Effects of tocotrienol on the molecular markers of aging skin.

Studies	Study design	Changes with tocotrienol treatment
Makpol et al. (2013)	Cells: Primary human diploid fibroblasts (HDFs) from foreskins of subjects, aged between 9–12 years	↑ COL1A1, COL3A1 genes
	Model: Oxidative stress induced by H_2_O_2_ for 2 weeks	↓ MMP-1, MMP-2, MMP-3, andMMP-9 genes
	Treatment: Pretreatment with TRF/Tocopherol (500 μg/ml; 100 μg/ml) at passage 4	
Makpol et al. (2010)	Cells: Normal skin fibroblast cell lines derived from young (21-year-old, YF) and middle-aged (40-year-old, MF) humans	↑ Telomerase activity (middle fibroblast), telomere length (young & old fibroblast)
	Treatment: γ-tocotrienol for 24 h before or after incubation with IC50 dose of H_2_O_2_ for 2 h	↓ Telomerase activity (young & old fibroblast)
		↔ telomere length (middle fibroblast)
Nakagawa et al. (2010)	Cells: Human keratinocytes (HaCaT) cells were incubated for 3 and 24 h in either SQ-OOH or γ-T3	↓ ROS generation, NF-kB activation, COX-2 mRNA expression, PGE2 production, interleukins
	Treatment: incubation for 3 and 24 h in either SQ-OOH or γ-T3. Various inflammatory markers were studied, such as interleukins, tumor necrosis factor-a, COX-2 and B-actin	
Makpol et al. (2012)	Cells: Primary human diploid fibroblast (HDFs) derived from foreskins of three 9- to 12-year-old boys after circumcision after consent was obtained	↑ BCL2AI mRNA
	Treatment: Young HDFs were exposed to prolonged low doses of H_2_O_2_, which mimics the oxidative stress *in vivo* to induce premature senescence state instead of acute induction with sublethal doses of H_2_O_2_	↓ Annexin V-FITC positive cells, cytochrome C, caspase-9 and caspase-3, BAX mRNA
Makpol et al. (2011)	Cells: derived from human skin fibroblast *Treatment*: cells were divided into groups of untreated control, hydrogen peroxide (H_2_O_2_)-induced oxidative stress (20 μM H_2_O_2_ exposure for 2 weeks), TRF treatment, and pre- and posttreatment of TRF to H_2_O_2_-induced oxidative stress	↑ COL I, COL III, collagen synthesis
		↔ COL IV

Abbreviation: ↓ decrease, inhibit or downregulate; ↑ increase, upregulate; ↔ no change; TRF, tocotrienol-rich fraction; COL, collagen; MMP, matrix metalloproteinases; ROS, reactive oxygen species; NF-κB, nuclear factor kappa B; COX-2, cyclooxygenase 2; PGE2, prostaglandin E2; SQ-OOH, squalene hydroperoxide; BCL2AI, B-cell lymphoma 2 AI; BAX, BCL2 associated X.

The earliest *in vitro* study was conducted by Makpol et al. (2010). The study compared the effect of γ-tocotrienol treatment on young and old human skin fibroblasts. γ-Tocotrienol was found to prevent telomere shortening by decreasing telomerase activity at concentrations of 80 µM for young fibroblasts and 40 µM for old fibroblasts. To evaluate the changes in collagen synthesis and skin-associated gene expression during aging, fibroblasts were subjected to oxidative stress induced by hydrogen peroxide (20 μM) for 2 weeks (Makpol et al., 2011). The researchers observed that oxidative stress reduced the expression of collagen genes, mainly COL I and COL III. Treatment with tocotrienol prior to the induction of oxidative stress benefited aging fibroblasts more compared to posttreatment, where the vitamin notably upregulated collagen production and collagen gene expression.

In a later study, the same group of researchers investigated the molecular mechanism of γ-tocotrienol in the stress-induced premature senescence (SIPS) model of fibroblast cells (Makpol et al., 2012). Oxidative stress was induced in the cells *via* exposure to low doses of hydrogen peroxide. Treatment with 1 µM γ-tocotrienol resulted in reductions in apoptosis markers, such as annexin v-FITC-positive cells, cytochrome C, and caspase-9 and caspase-3. The study concluded that γ-tocotrienol suppressed apoptosis by regulating the upstream apoptosis cascade, leading to the inhibition of apoptosis markers. In another study by Makpol et al. (2013), human diploid fibroblasts were treated with tocotrienol-rich fraction (TRF) (500 μg/ml) and tocopherol (100 μg/ml) either individually or in combination before being exposed to oxidative stress. The TRF in this study consisted of a tocotrienol and α-tocopherol mixture. Treatment with TRF alone or in combination with tocopherol reversed collagen degradation.

In a separate study, Nakagawa et al. (2010) tested the effects of γ-tocotrienol on inflammation induced by squalene hyperoxide in immortalized human keratinocytes. Cells were divided into two treatment groups receiving either squalene hyperoxide or γ-tocotrienol for the evaluation of inflammatory markers. Their results showed that γ-tocotrienol treatment reduced the production of interleukins, tumor necrosis factor-alpha (TNF-α), and cyclooxygenase 2 (COX-2), which were elevated by squalene hyperoxide.

Generally, the studies reported above showed that γ-tocotrienol upregulated the gene and protein expression of collagen, prevented collagen degradation and suppressed apoptosis in human fibroblasts undergoing senescence. It also prevented inflammation in human keratinocytes stimulated with oxidative stress from squalene hydroperoxide or hydrogen peroxide.

### Tocotrienol and ultraviolet damage

A total of 6 papers reported the effects of tocotrienol on UV-irradiated skin, of which one by Pedrelli et al. (2012) was performed on humans (Table 2). The study by Pedrelli et al. (2012) investigated the effectiveness of a topical formulation product enriched with vitamin E (10% tocopherols and 0.3% tocotrienols) on subjects diagnosed with photosensitivity. The formulation was applied to one side of the buttocks of the subjects for 30 min, while the other side served as the control. A total of 15 subjects were selected for further application of the vehicle cream on the forearm. They were then exposed to UV irradiation. The skin on the buttocks and forearm was tested for reactions, such as an extension of the lesion, erythema, edema, itch, and vesiculation. The results showed that the treated group had significantly lower skin reactions on the buttocks, except for erythema, than both the untreated and vehicle groups.

**TABLE 2:** Effects of tocotrienol on UV-damaged skin.

Studies	Study design	Changes with tocotrienol
Pedrelli et al. (2012)	Subjects: 30 humans aged 27–70 with a clinical history of photosensitivity	↓ Lesion, edema, itch, vesiculation
	Treatment: Topical combination containing active agents, tocopherols (at concentration of 10%) and tocotrienols (at concentration of 0.3%) for 30 min. The areas were irradiated with a 2-fold MED	↔ Erythema
Weber et al. (1997)	Animals: Female hairless mice	↑ α-tocotrienol, γ- tocotrienol after treatment
	*Treatment*: 20 µl 5% TRF in polyethylene glycol-400 (PEG), 20 µl PEG for 2 h. Half of the sites were exposed to UV-irradiation	↓ Total vitamin E after UV-irradiation
		↑ Vitamin E in TRF-treated compared to non-irradiated control
Yamada et al. (2008)	Animals: 5-week-old female hairless mice (*n* = 32 and *n* = 36)	↓ Sunburn in T-mix and T-mix + sesamin group
	Treatment: Experimental diet 50 mg/kg α-tocopherol, 229 mg/kg T-mix, 229 mg/kg T-mix & 2 g/kg sesamin for 6 weeks. Half of the mice were exposed to UVB light once daily for 7 days	
Traber et al. (1997)	Animals: Female hairless mice 8–12 weeks old	↑ Tocotrienols in skin tissue
	Treatment: Commercial chow diet containing α-tocopherol (30 ± 6 mg/kg, γ- tocopherol (10 ± 1 mg/kg diet), α-tocotrienol (3.1 ± 0.7 mg/kg diet), and γ-tocotrienol. 20 µl 5% TRF in polyethylene glycol-400 (PEG) for 2 h (Topical). The sites were exposed to UV-irradiation	↓ Total vitamin E after UV-irradiation
		↑ Vitamin E in TRF-treated compared to control
Brownlow et al. (2015)	Cells: Murine subcutaneous connective tissue fibroblasts	↑ Cell viability after UVB exposure
	Treatment: incubated for 24 and 48 h in non-emulsified propylenegycol based controls (Gen-PG and non-emulsified Tocomin-PG), empty vehicle and Gen-loaded prototype Tocomin NE	↓ Cutaneous irritation potential (with cosurfactant TPGS and LF68)
Shibata et al. (2010)	Cells: HaCaT cells were exposed to UVB	HaCaT cells: ↓ UVB-induced PGE2 (γ-T3), cyclooxgenase-2 (COX-2), interleukin (IL)-1β, IL-6, and monocyte chemotactic protein-1
	Treatment: cultured in test medium containing either γ-T3 or α-Toc for 2–24 h. UVB untreated cells were set as negative control	HR-1 hairless mice: ↓UVB-induced changes in skin thickness, COX-2 protein expression, and hyperplasia
	Animal: Female HR-1 hairless mice (10 weeks)	
	Treatment: divided into 4 groups: UVB-untreated/vehicle fed (Toc-stripped corn oil), UVB-irradiated/vehicle fed, UVB-irradiated/fed with 2.5 mg of α-Toc/day and UVB-irradiated/fed with 2.57 mg of RBT3 (0.09 mg of α-T3, 2.31 mg of γ-T3, 0.1 mg of δ-T3, 0.04 mg of γ-Toc, and 0.02 mg of δ-Toc)/day	

Abbreviation: ↑ increase, upregulate; ↓ decrease, inhibit or downregulate; ↔ no change; TRF, tocotrienol-rich fraction; UV-irradiation, ultraviolet-irradiation; UVB-induced PGE2, ultraviolet B-induced prostaglandin E_2_; COX-2, cyclooxygenase-2; IL-1β, interleukin-1β; IL-6, interleukin-6; RBT3, rice bran tocotrienol; α-Toc, alpha-tocopherol; α-T3, alpha-tocotrienol; γ-T3, gamma-tocotrienol; γ-Toc, gamma-tocopherol; δ-Toc, delta-tocopherol.

Three papers used female hairless mice to test the UV-protective effects of tocotrienols. Female hairless mice were treated with tocotrienol orally, topically or in combination before the induction of oxidative stress by UV irradiation. In a study by Traber et al. (1997), mice were fed a commercial chow diet containing α-tocopherol (30 ± 6 mg/kg diet), γ-tocopherol (10 ± 1 mg/kg diet), α-tocotrienol (3.1 ± 0.7 mg/kg diet), and γ-tocotrienol (7.4 ± 1.7 mg/kg diet). In addition, TRF was applied topically to the backs of the mice 2 h prior to irradiation. Elsewhere, Yamada et al. (2008) fed mice 4 types of experimental diets, namely, a vitamin E-free diet, an α-tocopherol diet (50 mg/kg), a T-mix (with 50 mg -tocopherol) diet (229 mg/kg) and a T-mix with 2 g/kg sesamin diet (229 mg/kg). T-mix is a combination of α-tocopherol (21.8%), γ-tocopherol (1.0%), α-tocotrienol (23.4%), and γ-tocotrienol (37.4%). Moreover, the sesamin diet is a mixture of lignans from sesame seeds. After 6 weeks, half of the mice were exposed to UV irradiation once daily for 7 days. In an early study by Weber et al. (1997), TRF (5%) was applied to the backs of mice for 2 h before exposure to UV irradiation.

The distribution of vitamin E was measured before and after treatment with TRF. The combination of oral and topical supplementation by Traber et al. (1997) showed that only α-tocopherol was found in the brain tissue. Other tissues also showed a higher content of α-tocopherol compared to other isomers. Conversely, tocotrienol levels were found to be higher than tocopherol levels in skin tissue. Exposure to UV irradiation significantly reduced the vitamin E content. However, the TRF-treated skin area contained higher vitamin E concentrations than the untreated control mice. Meanwhile, Yamada et al. (2008) found that the concentration of tocotrienol on the skin of the T-mix group was significantly lower than that on the skin of the α-tocopherol-fed group. The extent of sunburn was lowest in the T-mix + sesamin group, followed by the T-mix group. The α-tocopherol group showed a higher degree of sunburn, while the vitamin E-free group had the worst sunburn. These results show that the oral intake of tocotrienol helps to reduce skin damage caused by UVB. This photoprotective effect can be enhanced by the addition of sesamin.

The results from a study by Weber et al. (1997) using a topical formulation showed that treatment with TRF improved the antioxidant level of the skin (α-tocopherol 28 ± 16-fold, α-tocotrienol 80 ± 50-fold, γ-tocopherol 130 ± 108-fold, and γ-tocotrienol 51 ± 36-fold). However, the levels of all antioxidants were reduced significantly when the skin was exposed to UV irradiation. When compared to the control (non-irradiated polyethene glycol-400-treated group), the amount of vitamin E in the TRF-treated group was significantly higher even after irradiation. Based on these results, supplementation with tocotrienol, whether orally, topically or in combination, promoted the level of antioxidants in the skin.

Shibata et al. (2010) investigated the effect of γ-tocotrienol in inhibiting inflammation induced by UVB in both *in vitro* and *in vivo* models. The HaCaT cells used in this research were exposed to UVB and then cultured in test medium containing either γ-tocotrienol or α-tocopherol for 2–24 h. UVB-untreated cells were used as the negative control. In the animal model, female HR-1 hairless mice (10 weeks) were divided into 4 treatment groups: UVB-untreated/vehicle fed (tocopherol-stripped corn oil), UVB-irradiated/vehicle fed, UVB-irradiated/fed with 2.5 mg of α-tocopherol/day and UVB-irradiated/fed with 2.57 mg of rice bran T3 or RBT3 (0.09 mg of α-tocotrienol, 2.31 mg of γ-tocotrienol, 0.1 mg of δ-tocotrienol, 0.04 mg of γ-tocopherol, and 0.02 mg of δ-tocopherol)/day. The results showed that γ-tocotrienol treatment notably inhibited UVB-induced inflammatory markers compared to α-tocopherol in HaCaT cells. In HR-1 hairless mice, oral tocotrienol suppressed UVB-induced changes in skin thickness, COX-2 protein expression, and hyperplasia, while α-tocopherol resulted in no changes.

Brownlow et al. (2015) developed a nanoemulsified product from the TRF of palm oil to achieve an optimal nanoemulsion for skin photoprotection. To test the efficacy of the emulsion, fibroblasts originating from mice were divided into 3 treatment groups: non-emulsified propylenegycol-based controls, empty vehicle, and genistein-loaded (Gen-loaded) prototype Tocomin nanoemulsion, which contained the TRF of red palm oil. The study showed that TRF provided sufficient protection against UV irradiation, as it displayed the least reduction in antioxidant capacity while retaining a significant 78% of relative 2,2-diphenyl-1-picrylhydrazyl (DPPH) radical-scavenging activity. The Gen-loaded prototype showed potential in delivering antioxidant protection into skin layers without being partitioned, unlike many other topically applied cream formulations.

### Tocotrienol and pigmentation

A total of 4 papers reported the effects of tocotrienol on pigmentation during skin aging (Table 3). These included studies observing the anti-melanogenic properties of tocotrienol isomers using melanoma cells (Michihara et al., 2010; Yap et al., 2010; Makpol et al., 2014; Ng et al., 2014). Ng et al. (2014) induced melanoma cells with α-melanocyte-stimulating hormone before treating them with either δ-tocotrienol or arbutin for 24 h. Moreover, Makpol et al. (2014) induced melanogenesis using UVA for 6 days prior to treatment with either tyrostat, tocopherol, tocotrienol rich fraction or a mixture of all compounds. To study the effects of these treatments on pigmentation, the melanin content and tyrosinase enzyme activity were measured. Michihara et al. (2010) used different doses of δ-tocotrienol to study the effect on melanin content on B16 cells. Yap et al. (2010) performed several studies to understand the effects of tocotrienol isomers on tyrosinase activities in both human and animal samples. They also determined the interaction of tocotrienol with tyrosinase inhibitors and UVB-induced tyrosinase activity.

**TABLE 3:** Effects of tocotrienol and skin pigmentation.

Studies	Study design	Changes with tocotrienol treatment
Ng et al. (2014)	Cells: B16 melanoma cell line	↑ ERK cascade
	Treatment: δ-tocotrienol or arbutin for 24 h at various concentrations. The cells were stimulated with α-MSH before treatment	↓ Tyrosinase, TYRP-1, TYRP-2, Melanogenesis-related proteins
Makpol et al. (2014)	Cells: A primary culture of melanocytes was exposed to repeated doses of UVA for 6 days	↓ Tyrosinase, melanin synthesis, TYR, TYRP1, TYRP 2 genes
	Treatment: tyrostat, TRF or tocopherol alone or in combination	
Michihara et al. (2010)	Cells: B16 cells	↓ Melanin content, Tyrosinase, TRP-1, TRP-2 ( 25 or 50 µM δ-tocotrienol)
	Treatment: 25, 50 or 100 µM δ-tocotrienol for 48 h or 72 h	
Yap et al. (2010)	Cells: B16 melanoma cells	↓(Cells) Tyrosinase, melanin content
	Treatment: T3 isomers in a dose-dependent manner for 24 h	↓(Animal) tumor size, pigmentation of solid tumor
	Animal: Nude mice	
	Treatment: divided into control untreated group and γ-T3 treated group (100 mg/kg/day). The γ-T3 group were pretreated 1 week before B 16 cells inoculated onto the skin followed by 2-week post-treatment	

Abbreviations: ↑ increase, upregulate; ↓ decrease, inhibit or downregulate; ERK, extracellular signal-regulated kinases; TYR, tyrosinase gene; TRP/TYRP1/2, tyrosinase related protein 1/2; UVA, ultraviolet A; γ-T3, gamma-tocotrienol; α-MSH, alpha-melanocyte-stimulating hormone.

The results from all the studies showed that a tocotrienol mixture and its isomers were able to downregulate tyrosinase (TYR) and tyrosinase-related protein (TYRP/TRP) genes. Michihara et al. (2010) and Ng et al. (2014) reported that δ-tocotrienol effectively reduced the melanin content of the cells. Moreover, Makpol et al. (2014) observed decreased melanin content in cultures treated with TRF. Ng et al. (2014) found that δ-tocotrienol reduced melanin production similar to that produced by arbutin, which is known to inhibit melanosomal tyrosinase activity. These results indicate that δ-tocotrienol may act as a whitening agent.

Yap et al. (2010) adopted several designs in their study to investigate the effect of tocotrienol and pigmentation. In the first study, murine melanoma cells were treated with various tocotrienol isomers for 24 h. The results showed that α-tocotrienols at either 10 µM or 20 µM were unable to inhibit the proliferation of melanoma cells. Interestingly, only γ-tocotrienol, δ-tocotrienol, and TRF were found to significantly inhibit tyrosinase activity. Compared to individual isomers, TRF had the best efficacy against UV irradiation. Synergistically, γ-tocotrienol, δ-tocotrienol, and kojic acid boosted the inhibitory effect on tyrosinase levels.

### Tocotrienol and skin moisturization and wrinkles

Skin hydration and TEWL are indicators used to measure skin health in terms of moisture. The amount of water retained and water loss determine the moisturization status of the skin (Ijaz and Akhtar, 2020). Two clinical trials evaluated the effectiveness of tocotrienol-enriched cosmetic products in delivering moisturization (Table 4). Weber et al. (2003) tested the effect of a 14-day regimen on skin moisturization. The regimen consisted of 5% w/vol α-tocotrienol treatment followed by 10% benzoyl peroxide (BPO) treatment for 7 days each. BPO was used to increase lipid peroxidation and disturbance in the skin barrier (Weber et al., 2003). In another clinical trial, Ismail et al. (2009a) developed a whitening lotion enriched with tocotrienol and applied it to the skin of subjects for 6 weeks to assess its effects on skin moisturization.

**TABLE 4:** Effects of tocotrienol on skin moisture and wrinkles.

Studies	Study design	Changes with tocotrienol
Weber et al. (2003)	Subjects: 11 humans with skin types II and III, aged from 20 to 34 years	↑ Total vitamin E (pretreatment)
	Treatment: α-tocotrienol (5% w/vol) and control on the upper back for 7 days, addition of BPO (10%) treatment for another 7 days (topical)	↓ Total vitamin E after BPO treatment in control and α-tocotrienol treatment
		↔α-tocotrienol in α-tocotrienol treatment
		↑ TEWL after BPO treatment
		↓ MDA in α-tocotrienol treatment
Ismail et al. (2009b)	Subjects: 20 humans, aged between 23 and 49, in good health and free from skin diseases	↑ Skin hydration and lightness
	Treatment: Skin-whitening lotion enriched with tocotrienols for 4 weeks (topical)	↓ Melanin index
Ismail et al. (2009a)	Subjects: 12 humans	↑ Skin hydration
	Treatment: Tocotrienol-rich anti-wrinkle lotion for 60 days (topical)	↓Wrinkle, average skin roughness

Abbreviation: ↑ increase, upregulate; ↓ decrease, inhibit or downregulate; ↔ no change; BPO, benzoyl peroxide; MDA, malondialdehyde; TEWL, transepidermal water loss.

The study by Weber et al. (2003) found that the application of BPO reduced the content of vitamin E and increased TEWL significantly. Treatment with α-tocotrienol maintained the levels of α-tocotrienol and α-tocopherol in the stratum corneum (SC). Nevertheless, α-tocotrienol treatment failed to improve the TEWL that was increased by BPO, which is commercially used as an anti-acne topical treatment. The results of the study by Ismail et al. (2009b) showed that skin moisture was improved after 5–6 weeks of application with the self-developed whitening lotion containing tocotrienol. The skin-whitening effects were noticeable as early as 4–6 weeks.

Only one study reported on the effect of tocotrienol on skin wrinkles. In a separate study, Ismail et al. (2009a) studied the efficacy of tocotrienol as a component in an anti-wrinkle lotion. The lotion was applied on the forearm area of 12 subjects for 60 days. The results showed that skin hydration was improved, while the roughness of the skin and appearance of wrinkles were markedly reduced.

## Discussion

### Tocotrienol and skin aging

Molecular mechanisms that are involved in skin aging include oxidative stress, mitochondrial DNA mutations, DNA damage and telomere shortening (Lee, 2021). Among these factors, oxidative stress has been widely studied as one of the main causes of skin aging. Oxidative stress also activates inflammatory pathways through the activation of NF-κB (Chaiprasongsuk and Panich, 2022), subsequently triggering the production of inflammatory cytokines, such as IL-6, TNF-α, and COX-2 (Zhuang and Lyga, 2014; Bang et al., 2021). Both COX-2 and TNF-α are believed to contribute to the inflammatory effect in keratinocytes (Nakagawa et al., 2010). The accumulation of reactive oxygen species (ROS), such as hydrogen peroxide, that surpass the antioxidant capacity of cells will consequently result in oxidative stress. Based on this mechanism, many antioxidants, including tocotrienol, have been studied to combat skin aging. Both extrinsic (UV radiation) and intrinsic factors contribute to skin aging *via* inflammatory processes.

Aging negatively affects the skin in terms of the development of wrinkles, loss of elasticity and loss of hydration. Zhang and Duan (2018) suggested that aging results in the reduction of procollagen I synthesis due to the downregulation of TGF-β/Smad signaling. The overproduction of ROS as a result of UV radiation also induces downstream signaling, which activates mitogen-activated protein kinase (MAPK) and nuclear factor-κB (NF-κB). The activation of NF-κB leads to a decrease in collagen production, while the level of the MMP gene increases (Zhang and Duan, 2018).

In addition, the increase in ROS would also affect telomere length. Specifically, telomere shortening results in premature cell death or senescence. Naturally, telomeres decrease in length through cell division. Telomerase is an enzyme with crucial responsibility, as it provides telomere repetitions at the end of the telomere to stop shortening. As reported by Makpol et al. (2010), oxidative stress affects telomere length and telomerase activity in fibroblasts from young, middle-aged, and old subjects. The introduction of γ-tocotrienol helped to preserve telomere length and decreased telomerase activity in old fibroblasts. γ-Tocotrienol could also reduce ROS generation, NF-κB, COX-2 mRNA expression, PGE_2_ production, and interleukins (Makpol et al., 2010; Nakagawa et al., 2010). The combination of these effects resulted in improved skin health. Moreover, TRF was found to increase collagen gene synthesis and decrease MMP genes (Makpol et al., 2010; Makpol et al., 2013) *via* the upregulation of COL I and COL III, both of which contribute to the elevation of collagen production in the skin. Collagen content in the skin is mainly produced in fibroblasts, which function to provide elasticity (Zhang and Duan, 2018). Matrix metalloproteinases (MMPs) are enzymes that determine the production of collagen in the skin. High MMP activity affects the structure of the skin, as it impairs the synthesis of collagen (Makpol et al., 2010).

Low-grade inflammation, or inflammageing, is known as the major cause of age-related health issues, such as metabolic diseases, cardiovascular diseases, cognitive decline, bone fragility, and the decline in skin health (Zhuang and Lyga, 2014; Fougère et al., 2017; Zhang and Duan, 2018). Several studies have been performed to understand how inflammatory activities could affect skin health. UV exposure increases oxidized lipids in cells, thus triggering inflammation. Inflammation stimulates macrophages to eliminate damaged cells and oxidized lipids from the system. Subsequently, MMPs are released to degenerate the extracellular matrix. Overexposure to UV radiation causes an accumulation of oxidized lipids that can overwhelm the action of macrophages and lead to the release of proinflammatory cytokines and ROS (Zhuang and Lyga, 2014).

**FIGURE 2:**
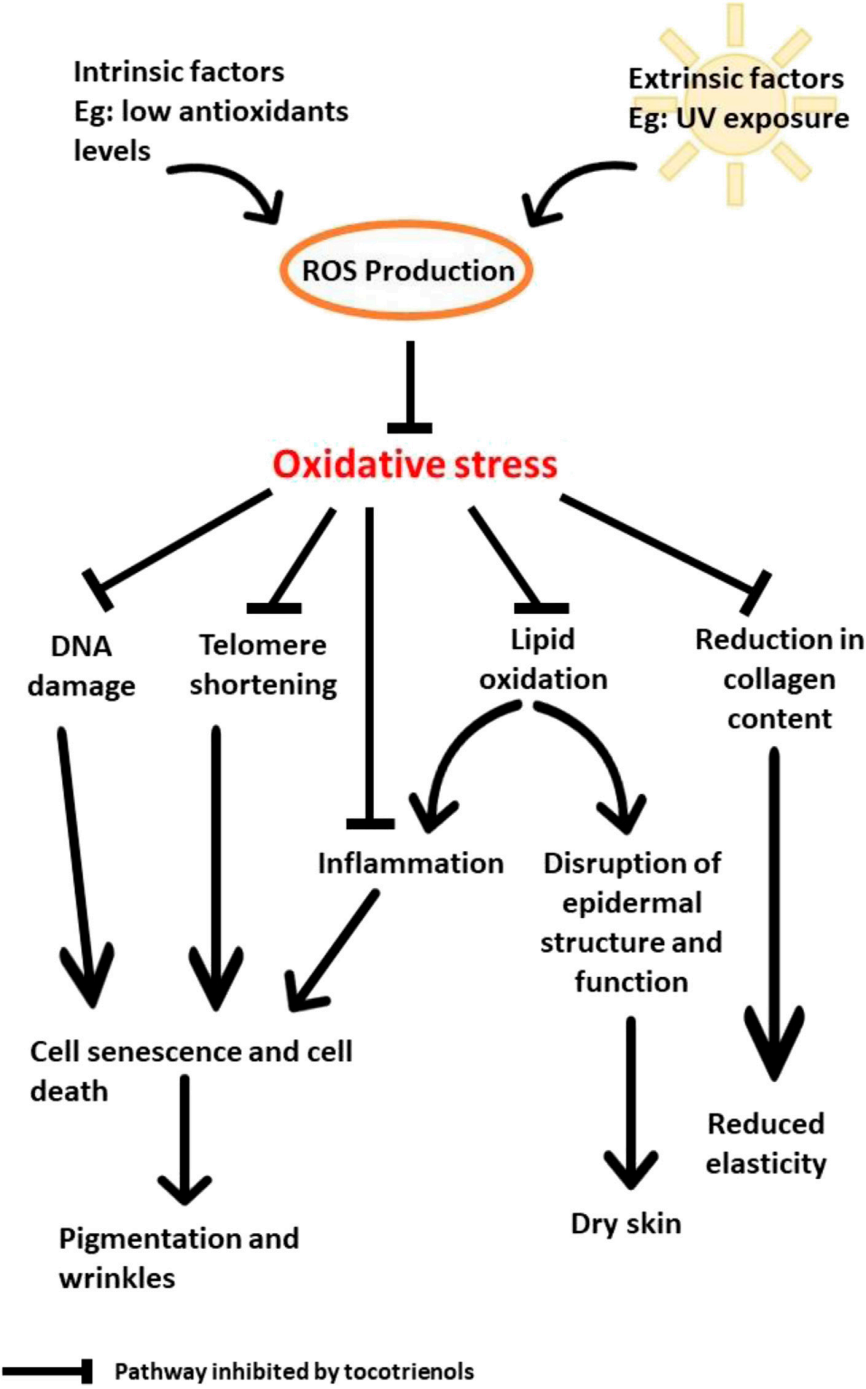
The skin aging pathways inhibited by tocotrienols.

Based on the reported findings, UV is undoubtedly the main factor that contributes to oxidative stress and the deterioration of the physical appearance of skin by increasing the production of ROS. The accumulation of ROS leads to the oxidation of DNA and lipids in addition to the shortening of the telomeres and disruption of collagen synthesis. As a result, the skin becomes dry, less elastic, more pigmented, and wrinkly. Due to its potent antioxidant properties, tocotrienols can help to prevent and reduce the damage caused by UV irradiation by inhibiting DNA damage, lipid oxidation, telomere shortening, and the reduction in collagen content (Figure 2). At low levels of antioxidants, oxidative stress causes premature senescence, and death of skin cells that lead to pigmentation and wrinkling. In addition, oxidation of lipids will occur in the epidermis. This disrupts epidermal structure and function that result in dry skin. Oxidative stress also obstructs collagen synthesis and lead to reduced skin elasticity.

### Tocotrienol and ultraviolet radiation

UV radiation can be divided into three types: UVA, UVB, and UVC. Only UVA and UVB radiation can penetrate our ozone layer. These two types of rays were reported to have different permeability rates in human skin. UVA radiation was reported to have a deeper penetration ability, as it can have negative impacts on the cellular and extracellular structure of the dermis layer. Conversely, UVB radiation has a lower penetration rate, as it can only penetrate up to the epidermis layer. Together with blue light, UVA radiation becomes a threat to our skin in terms of inducing skin aging, including causing wrinkles, reducing hydration, and promoting skin cancer (Hossain et al., 2021). Specifically, 80% of skin aging reportedly results from Sun exposure (Zhang and Duan, 2018). Apart from directly harming our skin, UV radiation also causes damage to our DNA in two ways: by direct DNA absorption, which directly mutates DNA, and by increasing ROS production in the skin through pigments produced by organisms, which subsequently cause oxidative stress-induced apoptosis or cancer development (Chaiprasongsuk and Panich, 2022).

MAPK phosphorylation and ROS production in human keratinocyte cells are suppressed *in vitro* with γ-tocotrienol treatment (Shibata et al., 2010). MAPK proteins, such as ERK and p38, play an important role in cellular development. COX-2 or cyclooxygenase-2 is the key enzyme that facilitates the production of PGE_2_ from arachidonic acid (Black et al., 2008). COX-2 expression in cells is triggered by the MAPK signal (Chen et al., 2001). Exposure to UVB induces the accumulation of ROS in cells, which further activates NF-κB. In addition, ROS production can also activate MAPK signaling molecules (Shibata et al., 2010). The downregulation of PGE_2_ is crucial to stop the inflammatory effects caused by UVB radiation, as it acts as the main modulator that activates the release of other inflammatory factors, such as interleukins. Shibata et al. (2010) proved that γ-tocotrienol can suppress PGE_2_, COX-2, and interleukins *in vitro*. When tested orally in hairless mice, tocotrienol suppresses COX-2 protein expression and reduces skin damage. In addition, Ahn et al. (2007) reported that γ-tocotrienol can inhibit the NF-κB activation pathway, which further decreased the expression of antiapoptotic gene products, leading to the downregulation of various gene products. These findings suggest that tocotrienol provided a protective effect against UV irradiation.

### Tocotrienol and pigmentation

Skin pigmentation naturally occurs as a result of UV exposure. UV exposure increases measures of pigmentation, such as melanin and erythema. The skin produces melanin as a defense mechanism against the harmful effects of UVB, UVA, and blue visible light irradiation (Solano, 2020). Within the skin, melanin is produced by melanosomes, which are a type of lysosome (Zhu et al., 2020). They scavenge ROS in reaction to oxidative stress. UV radiation exposure also provokes skin inflammation by producing inflammatory cytokines from epidermal keratinocytes and dermal fibroblasts. Cytokines trigger the activity of melanocytes through various markers, such as interleukins (Hossain et al., 2021). In addition to melanin, the activity of tyrosinase is also used as an indicator of skin pigmentation (Yap et al., 2010). Tyrosinase is the key enzyme involved in the melanogenesis biochemical pathway. Exposure to UV radiation activates tyrosinase, leading to an increase in melanin production through melanogenesis (Yap et al., 2010; Solano, 2020).

The production, distribution, type, and quantity of melanin determine the effect of UV radiation on skin pigmentation. The synthesis of melanin through the melanogenesis pathway is regulated by the L-tyrosine enzyme tyrosinase. This enzyme is stabilized by tyrosinase-related protein (TRP) 1 and 2 in the process of melanin formation. TRP is also involved in the synthesis of melanosomes and protection against oxidative stress (Hossain et al., 2021). Tocotrienol is believed to help reduce skin pigmentation through the downregulation of the melanogenesis pathway (Ng et al., 2014). However, research on the role of tocotrienol in skin pigmentation in animal models remains limited. γ-Tocotrienol was found to reduce pigmentation in skin tumors induced in nude mice (Yap et al., 2010). Based on *in vitro* studies, δ-tocotrienol is believed to be able to downregulate the production of melanin (Michihara et al., 2010; Ng et al., 2014). Makpol et al. (2014) also reported that TRF in combination with tocopherol effectively reversed the damage caused by UV-induced melanogenesis *in vitro*. These findings indicate the promising role of tocotrienol in reducing melanin production during aging. Future studies should elucidate the role of each of its isomers on skin pigmentation to identify the best isomer(s) to be used for further *in vivo* testing.

### Tocotrienol and skin moisturization

The application of moisturizers is essential to help maintain hydration and reduce water loss of the skin. The outermost layer of the epidermis, the SC, is the targeted skin surface of most moisturizers since it contains natural moisturizing factors (NMFs) and lipids that are involved in maintaining skin moisture (Vaz et al., 2019). The SC acts as a skin barrier due to its occlusive effects formed by the lipid bilayer matrix (Foster et al., 2020). In addition, epidermal proteins, such as aquaporin-3 (AQP3), also help to retain water content in the SC (Verdier-Sévrain and Bonté, 2007). The efficiency of SC to sustain water content could be impaired under certain conditions. Prolonged exposure to the Sun, wind and low humidity can cause damage to the structure of lipids and disrupt epidermal differentiation and collagen synthesis, leading to inflammation and water loss (Vaz et al., 2019). In addition, aging can also impair skin moisture due to modifications of the fatty acids that hold the structure of the hydrophilic film (Sonneville-Aubrun et al., 2018).

Vitamin E is widely used in skincare and cosmetic formulations due to its antioxidant and liposoluble properties. Researchers have been studying the efficacy of using different types of vehicles, such as lipid nanoparticles, nanoemulsions, humectants, and chitosans, to transport and improve the release of vitamin E into the epidermis due to its inherent instability and short lifespan (Chen and Heh, 2000; Yenilmez et al., 2011; Vaz et al., 2019; Ijaz and Akhtar, 2020). Interestingly, combinations of vitamin E with these vehicles have shown more positive results in increasing skin hydration and reducing TEWL compared to using the vitamin alone. These vehicles are suggested to be able to form an occlusive film on the skin surface that prevents water loss, in addition to increasing the permeation of vitamin E. Although many studies have shown the positive effect of vitamin E in maintaining skin moisture, the cellular mechanism involved remains unclear. Since the SC is closely related to the maintenance of skin moisture, the role of vitamin E treatment in the structural changes may warrant further exploration. Moreover, many studies have focused on the use of tocopherol rather than tocotrienol in maintaining skin moisture. Comparing the effects of different vitamin E isomers on the skin moisture level in a similar animal model in the future will be interesting.

### Tocotrienol and wrinkles

The physical features of wrinkles caused by intrinsic and extrinsic factors are different. Extrinsic aging caused by UV exposure and smoking leads to deep, coarse wrinkles, while intrinsic aging produces fine wrinkles over time (Langton et al., 2010). Wrinkles develop due to extracellular matrix destruction and skin inflammation (Bae et al., 2010). UV radiation causes skin cells to undergo inflammatory responses *via* the increased production of various inflammatory mediators. Proinflammatory mediators activate various MMPs that result in abnormal matrix degradation and the accumulation of non-functional matrix components in the dermal and epidermal compartments (Bae et al., 2010). The extracellular matrix consists of collagen, elastin, and proteoglycans that are present in the dermal matrix. These structures give strength and resiliency to the skin (Song et al., 2020). Therefore, abnormal matrix degradation leads to wrinkle formation. In addition, increased MMP expression also reduces collagen synthesis and accelerates collagen degradation, which weakens the skin matrix (Song et al., 2020).

To date, limited studies have examined the effect of vitamin E on skin wrinkles. Ismail et al. (2009b) reported a reduction in wrinkles and roughness after the application of tocotrienol-rich anti-wrinkle lotion. However, no discussion on the mechanism of action of tocotrienol was included in the report. Therefore, the effect of vitamin E, especially tocotrienol, on wrinkles cannot be concluded.

## Conclusion

Tocotrienols are known antioxidants that have been incorporated into skincare products due to their antiaging properties. Available data suggest that oral and topical treatment with tocotrienols can delay skin aging by decreasing inflammation, melanin accumulation, and filtering UV exposure. However, reports on the effect of tocotrienols on wrinkle formation and moisture content of the skin are limited. Thus, more studies are necessary to establish the mechanism of action by which tocotrienols affect these measures. Since the bioavailability of tocotrienols is low, future studies should focus on improving the formulation of treatments to enhance their uptake by the skin. In addition, studies should compare the efficacy of oral and topical tocotrienol treatments in aging skin so that treatment route and dosage of tocotrienols can be optimized for anti-aging effects.
